# Supplementary material for: Systematic review of animal-based indicators to measure thermal, social, and immune-related stress in pigs
Source: PLoS One. 2022 May 5;17(5):e0266524. doi: 10.1371/journal.pone.0266524 (PMC9070874; doi:10.1371/journal.pone.0266524)
Supplement: Attachment — Submitted filename: RebuttalLetter-PLOSONE2021.docx [file pone.0266524.s004.docx]

July 9, 2021

Cerdanyola del Valles, Barcelona, Spain.

Dear editors,

We thank the associate editors for their comments on the manuscript and we have edited the manuscript to address their concerns.

In particular, the description about who performed the literature searches for the systematic review (page 5, lines 102 – 104) and we clarify the data extraction and quality assessment protocol (page 6, lines 127 – 130). Furthermore, we explain the strategies to reduce and control publication bias risks (page 6, lines 130 – 133).

We believe that the manuscript is now suitable for publication in PLOS ONE. We appreciate the opportunity to publish our work in your journal.

Raúl David Guevara Ballesteros

Associate researcher from the Animal Welfare Education Center (AWEC).

On behalf of all the authors.
