# Supplementary material for: Systematic review of animal-based indicators to measure thermal, social, and immune-related stress in pigs
Source: PLoS One. 2022 May 5;17(5):e0266524. doi: 10.1371/journal.pone.0266524 (PMC9070874; doi:10.1371/journal.pone.0266524)
Supplement: Attachment — Submitted filename: ResponseLetter 03-03-22.docx [file pone.0266524.s006.docx]

**Revision Note to manuscript PONE-D-21-22028**

We thank the editor for your valuable comments and invested time on reviewing our manuscript. We have taken them into consideration with the aim of fulfilling the information provided, contributing to clarify the conclusion section and the grammar of the manuscript. On the other hand, please, find our answers to the comments posted below. Those are referred as the authors’ response in red (AR:).

**Editor’s comments:** Authors claim that the manuscript has been amended according to our comments, but in fact no restructuring was done at all, and no new information was included, with the revised version being practically the same as the original one, which is disappointing. Besides the argumentation for the reviewers, authors should make an effort to improve the paper, (which is the goal of the review process) for instance by highlighting the novel aspects of the work (possibly in the objectives and in the conclusion section which should be newly written, see last sentence below) or adding some data as suggested by reviewer 2.
Authors’ Response (AR): We acknowledge the comments of the editor about the reviewers’ comments on the structure of the literature review. We tried to justify the review structure used in the manuscript, with detailed examples of the novelty of the structure relative to the reviewees available on the literature. Additionally, we have made the suggested changes to increase the clarity of the manuscript. For instance, we have clarified cytokines method of measurement in tissues (Lines 305-309), we added information regarding hair cortisol measurement and the possible weakness of fecal samples as less- invasive method (Lines 603 -607), and we have included information about the indicator direction change (it increase, decreases, or equals the value relative to the control). We believe that this may help the reader understanding what is being actually being measured, and what is representing the indicator value (Lines 363-365, 383-286, 395-397, 431-434, 452-454, and 477-478 for the thermal stress model; Lines 534-538, 566-568, 582-584, and 627-628 for the social stress model; Lines 663-666, 679-680, 687-690, 694-696 for the immune-related stress model). We think that the comments provided by the reviewers and the editor have helped to generate a fine piece of literature that accomplishes to show the trends of measurement methods and indicators to monitor stress in pig production.

Besides, the manuscript still needs a very deep revision of English language and writing before it can be accepted for publication. Some examples (lines correspond to the submitted manuscript with track changes) are listed below, but this is not a comprehensive list, please revise the whole text

Lines 41-44: this sentence lacks a verb?

Lines 70-71: this sentence is weird, please rewrite

Line 82: change "de" to "the"

Line 98: peer-reviewed

Line 114: counted

Line 118: methodologies for measurement of indicators

Line 124: impact of the stress ON the animals

Lines 187-188: something is lacking

Line 196: physiological markers

Lines 208-209: blood sampling is not a methodology for glucose measurement, it is just the source

Lines 199-225: I don't see the need of subheadings for the presentation of these results, same for the other types of stress model

Line 240: The term Physiology" can not be used as adjective

Line 242: "assessed with the assessment" please rewrite

Line 245: effect ON

Line 246: change "includes" to "include"

Line 263: with 162 papers reporting this type of indicator?... please phrase a little bit....

Line 266: change "in" to "for"

Line 269: more reiterated methodology

Line 298: delete "assess"

Line 304: which supplementary file?

Lines 306-307: delete "."

Line 312: raisings?

Line 317: "Cytokines` gene expression measurement could be fit into the physiological markers category, but it was decided to keep it at the blood chemistry group to avoid confusion. Separation of the blood chemistry from the physiological markers was decided due to the variety of markers that are measured at blood".

Measurement of citokine....

Change "could be fit" to "could fit"

Line 346: causes the stress

Lines 353-357: repetition with intro

Line 383: Particularly, the increase of the index relative to animals in thermo neutral conditions. Which index, you haven't mentioned a particular one, do you mean each index?

Line 386: what is black globe temperature?

Lines 394-396: generation of notifications? what do you mean?

Line 414: has been

Line 419: delete "it"

Line 430: will depend

Line 432: production units / generate

Line 435: because of their LACK OF invasiveness

Line 439: On the other hand

Line 443: induce pain to the animal

Line 452: change stratus to status

Line 471: different kinds of studies

Line 488: are still considered

Line 512: change thirsty to thirst

Line 532: these social orders

Line 538: social stress affects

Line 539: reduces and risks

Line 544: these indicators

Line 546: "The frequency increase " Change to increase in frequency of... has been detected

Line 571: these indicators

Line 572: depends

Line 578: "This methodology assumes that animals more aggressive will begin more agonistic encounters" Change to more aggressive animals

Line 614: "hair cortisol measurements has" change to "have"

Line 621: than cortisol

Line 640: consequences on the integrity

Line 648: amply?? do you mean widely?

Line 669: delete "organism of the"

Line 673: "Biochemical indicators rises measurements" does not make sense. Do you mean rised measurements of biochemical indicators?

Line 696: delete" for its detection"

Line 722: require

Line 723: involve

Line 779: "The use of holistic approaches and the possible complementary of indicators ..." complementary of indicators does not make sense. Do you mean complementarity?

AR: We appreciate the time and commitment of the editor to review the language grammar. We made all changes suggested by reviewers’ comments. Additionally, a language review was performed by a professional scientific literature editing service. The language review track changes file and the language review certificate are uploaded with the manuscript.

The text included in the conclusions section does not fit in that heading but is in fact a joint discussion for the different types of stress and their indicators. This part should me moved to discussion under a proper heading, and a new conclusion section should be written

AR: We thank the editor for your valuable comments about the discussion and conclusions sections. We have taken them into consideration and the amendments have been done to the text aiming to clarify the conclusion section of the manuscript. The new conclusion section can be found at Lines 773 – 791.

We hope that experts take into consideration our amendments, clarifications and reasoning for further evaluations of the manuscript.

As mentioned, we thank all valuable comments and suggestions addressed by reviewers to improve the manuscript and their dedication and willingness to refine the quality of our systematic review.
